# In silico investigation of thiazole–semicarbazide hybrids as dual GSK-3β/Tau inhibitors for Alzheimer’s disease

**DOI:** 10.1038/s41598-026-55932-9

**Published:** 2026-06-03

**Authors:** Dileep Kumar, Vinayak Walhekar, K. Mangala Shenoy, Suvarna G. Kini

**Affiliations:** 1 Department of Pharmaceutical Chemistry, Manipal College of Pharmaceutical Sciences, Manipal Academy of Higher Education, Manipal, Karnataka 576104 India; 2 Department of Pharmaceutical Chemistry, School of Pharmacy and Research, Dnyaan Prasad Global University, Pimpri Pune, Maharashtra 411018 India

**Keywords:** Alzheimer’s disease, GSK-3β, Tau, Molecular dynamics, Molecular docking, ADMET, Biochemistry, Chemistry, Computational biology and bioinformatics, Drug discovery

## Abstract

AD is a widespread and debilitating neurodegenerative disorder, and existing treatments have demonstrated limited efficacy, emphasizing the need for novel therapeutic strategies. This study focused on the design of drug-like molecules with enhanced efficacy and minimized side effects by application of structure-based scaffold hopping and molecular hybridization strategies. Molecular docking was carried out on Glide module; Molecular dynamics simulation of 500 ns was executed employing Desmond and ADMET prediction was achieved by QikProp modules of Schrödinger. Through molecular docking studies targeting the GSK-3β and Tau enzymes, the compounds **DVK5 and DVK11** were identified as promising inhibitors, showing favorable interactions within the active sites of these proteins, with docking energies of **− 9.863 and – 8.994 kcal/mol**, respectively. Molecular dynamics simulations further revealed that the **DVK5 and DVK11** complexes exhibited stable interactions within the active sites of GSK-3β and Tau throughout a 500 ns simulation. Additionally, in silico ADMET analysis demonstrated that **DVK10** exhibited an excellent human oral absorption rate of **75.175%**, outperforming other compounds in the series. These findings strongly suggest the potential of **DVK5 and DVK11** as dual inhibitors of GSK-3β and Tau, offering a basis for future drug development studies for the development of new lead compounds for AD treatment.

## Introduction

Alzheimer’s disease (AD) is a progressive neurodegenerative condition and the primary cause of dementia, accounting for 70–95% of cases worldwide^1^. It is characterized by a gradual decline in cognitive function, memory impairment, and behavioral disturbances, ultimately culminating in severe disability and mortality. Affecting over 66 million individuals globally and with a new diagnosis occurring every 4.2 s, AD represents an escalating public health concern. In 2024, an estimated 6.7 million Americans aged 68 and older were living with AD, with the risk doubling approximately every five years beyond this age^2^. On a pathological level, AD is defined by the accumulation of amyloid-beta plaques and Tau protein tangles, which lead to synaptic dysfunction, neuronal loss, and brain atrophy^3^.

The exact etiology of Alzheimer’s disease is multifaceted, but genetic factors, including mutations in APP, PSEN1, PSEN2 and the presence of the APOE ε4 allele, are recognized as significant contributors to risk^4^. The economic impact of the disease is profound, with global costs related to dementia exceeding $2.0 trillion in 2021 and projected to escalate to $2.8 trillion by 2040. While current treatments primarily address symptoms, the focus of research has shifted toward the development of disease-modifying therapies targeting amyloid and Tau pathways^5^. Additionally, attention is growing around novel therapeutic targets such as neuroinflammation, oxidative stress, and mitochondrial dysfunction, underscoring the pressing need for innovative and effective strategies^6,7^.

Glycogen synthase kinase 3β (GSK-3β) is a serine/threonine kinase integral to numerous cellular functions, including metabolism, cell differentiation, and apoptosis. Its dysregulation is strongly implicated in the pathogenesis of AD^8^. GSK-3β plays a pivotal role in Tau protein phosphorylation, leading to its hyperphosphorylation and subsequent aggregation into neurofibrillary tangles, which are key contributors to neuronal dysfunction and degeneration. Additionally, GSK-3β influences amyloid precursor protein (APP) processing, promoting the generation of amyloid-beta (Aβ) peptides-a defining feature of AD pathology^9^. The kinase also modulates neuroinflammatory pathways by regulating microglial activation, further amplifying neuronal damage. Dysregulated GSK-3β activity has been associated with neuronal apoptosis and cell loss, while its effects on synaptic plasticity adversely impact cognitive functions such as learning and memory^10^. As a result, GSK-3β has emerged as a compelling therapeutic target, with ongoing research exploring inhibitors aimed at reducing Tau hyperphosphorylation, altering APP processing, and mitigating neuroinflammation to potentially slow disease progression^11^.

Tau protein is integral to the progression of AD, shifting from its normal role as a microtubule-stabilizing protein in neurons to forming harmful neurofibrillary tangles^12^. Under healthy conditions, Tau supports the structural stability of microtubules and aids in intracellular transport^13^. In AD, however, Tau becomes abnormally hyperphosphorylated, a process influenced by factors like Aβ accumulation, which disrupts cellular signaling and creates an enzyme imbalance^14^. This leads to the formation of insoluble Tau aggregates that disrupt neuronal function and drive cognitive decline^15^. Although Aβ buildup is considered an early event in AD, Tau abnormalities more closely align with the severity of cognitive impairment, making it a key target for therapy^16^. Efforts to address Tau pathology include preventing its aggregation, employing immunotherapies to clear defective proteins, and stabilizing microtubules to restore normal cell function^17^. While there are challenges in developing effective treatments, these strategies hold potential for mitigating neurodegeneration in AD.

Aβ accumulation can stimulate GSK-3β activity, which then drives the hyperphosphorylation of Tau protein, creating a vicious cycle that accelerates neurodegeneration. This relationship highlights GSK-3β as a promising therapeutic target, as its inhibition could simultaneously reduce Aβ production and Tau abnormalities. Such interventions have the potential to slow or halt the cognitive decline associated with AD by addressing two key pathological mechanisms^18,19^.

Concurrent targeting of GSK-3β and regulation of Tau leads to a complex physiological outcome with clear relevance to neurodegenerative disorders such as Alzheimer’s disease. At the neuronal level, this strategy limits Tau hyperphosphorylation by suppressing kinase activity while also reducing the availability of Tau itself, thereby restricting aggregation, preserving microtubule integrity, and supporting efficient axonal transport and synaptic stability. In parallel, inhibition of GSK-3β can enhance pro-survival signalling pathways, while reduced Tau-associated toxicity alleviates oxidative stress and mitochondrial impairment, collectively promoting neuronal viability. Despite these benefits, the approach requires careful consideration, as GSK-3β is involved in multiple physiological processes, including metabolic regulation and intracellular signalling, and its sustained inhibition may lead to systemic imbalances such as altered glucose handling. Likewise, Tau contributes to cytoskeletal organization, and excessive downregulation may compromise neuronal plasticity and adaptability. Thus, while dual modulation can effectively reduce pathological burden and improve structural stability, excessive suppression may limit the dynamic behaviour necessary for normal neuronal function, indicating that a balanced, fine tuned approach is more appropriate than complete inhibition.

**Fig. 1:**
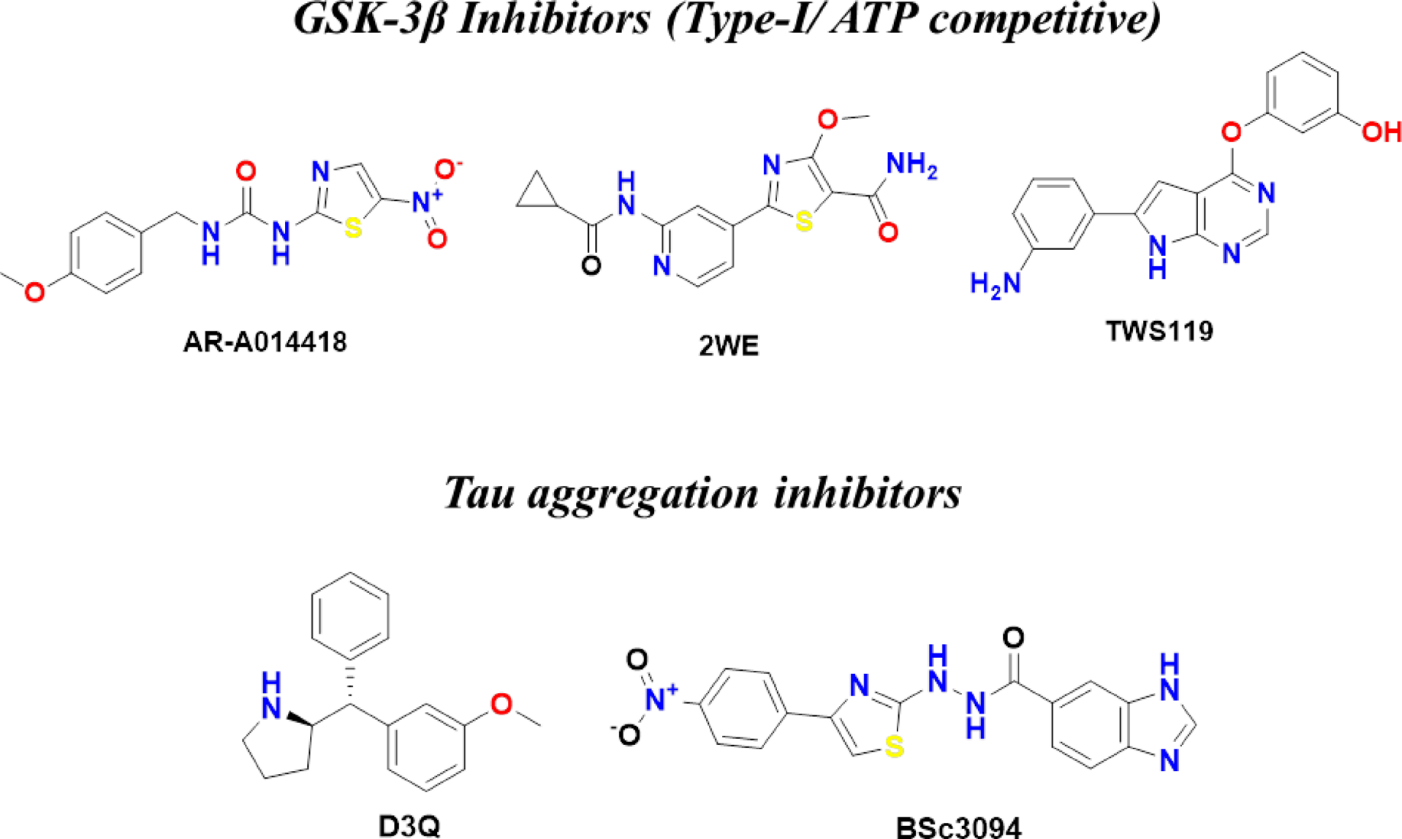
Reported inhibitors of Tau aggregation and GSK-3β.

Various inhibitors targeting of GSK-3β and Tau are registered separately but to date there is no drug like moiety or a drug is reported that simultaneously block the aforementioned targets^20,21^. To address AD, researchers are focusing on designing novel dual inhibitors targeting specific kinases using structure-based drug design methods. These inhibitors are being assessed for their pharmacokinetic properties, including ADMET, through computational studies. This approach aims to identify lead compounds with the potential for further development into effective therapeutic agents for combating AD (Fig. 1).

## Molecule crafting strategy

The design and development of innovative multitargeted drugs can be achieved through two primary strategies: integrated and hybrid ligands. Recent research has focused on the incorporation of thiazole, piperidine and pyrrolidine to create hybrid ligands, which offer a unique opportunity to develop compounds with enhanced therapeutic efficacy. By merging two pharmacologically active components, the objective is to leverage their synergistic effects, thereby expanding the therapeutic potential and action spectrum of the resulting molecules.

To support this hypothesis, the current study involved designing various molecules using hybridization and scaffold hopping techniques. The designed compounds include piperidine, pyrrolidine, and thiazole hydrazide carboxymide-linked acetamide derivatives, which are anticipated to exhibit dual selectivity targeting Tau and GSK-3β. Following this molecular design approach, a total of sixteen compounds were synthesized, categorized as **DVK 1–9 (Piperidine)** and **DVK 10–16 (Pyrrolidine)**. These compounds feature substituted at the 4^th^position of the tail end phenyl ring, including -H, -F, -Cl, -Br, -NO_2_, -CF_3_, 3,4-ClF, and 3,4-ClCF_3_.

**Fig. 2:**
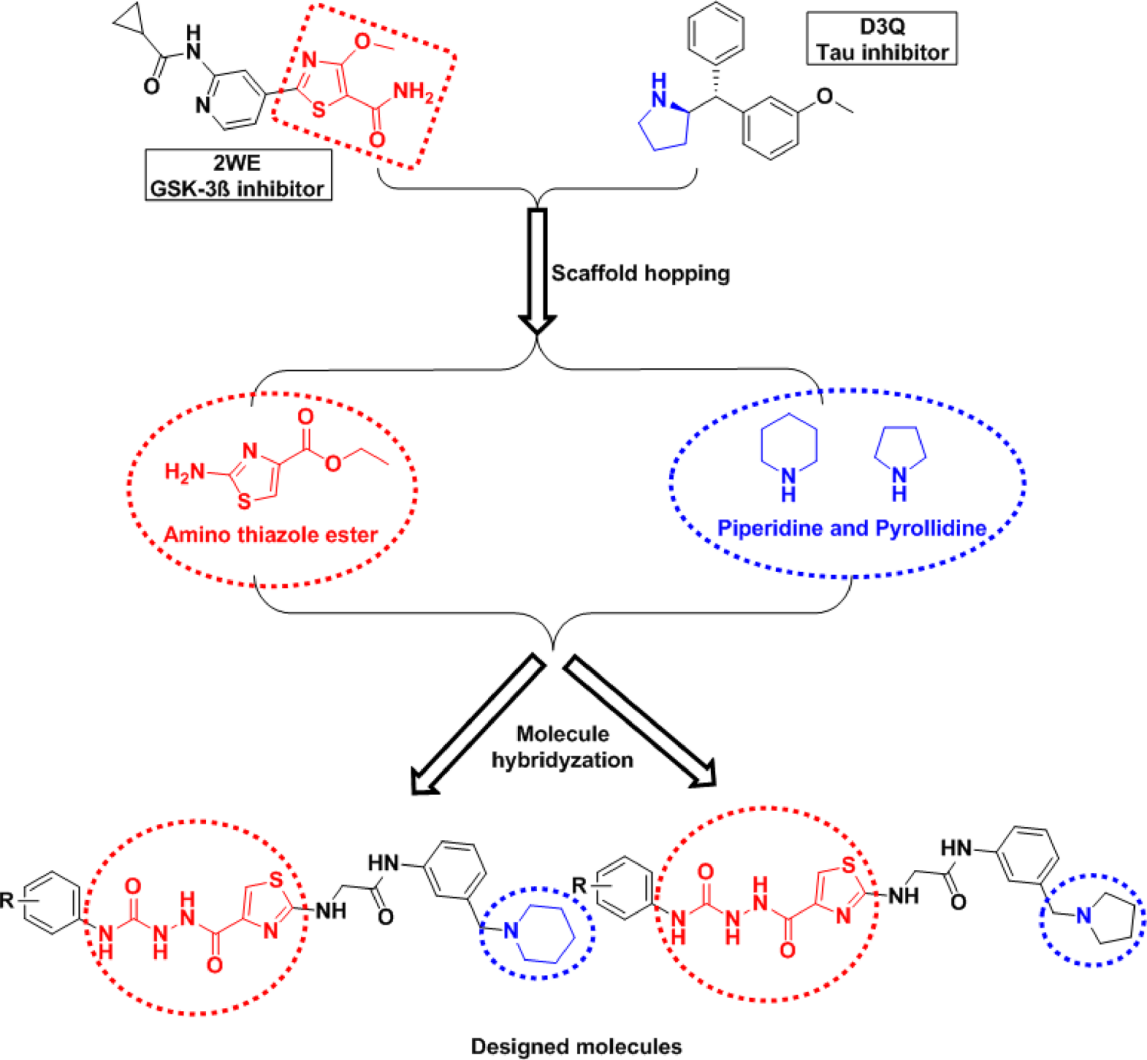
Crafting approach of dual inhibitors DVK linked GSK3β and Tau pharmacophores Piperidine, Pyrrolidine and Thiazole fragments by utilizing scaffold hopping and molecule hybridization approaches.

Given the observed liver toxicity and high molecular weights associated with piperidine and pyrollidine-based molecules, a fusion strategy was employed to strike a balance between multitarget inhibitory activity and improved physicochemical properties. This strategic approach aims to enhance the overall efficacy of the designed compounds while minimizing potential adverse effects (Fig. 2).

## Material and methods

### Molecular docking analysis

Molecular docking scrutiny was employed to recognize interconnections of ligand with the active pocket amino acids of macromolecule and conformations that finally result in docked complex solidity. Ligand preparation was conducted using the LigPrep module of GLIDE software (version 6.6) within Maestro version 14.0.134. To generate ligand conformers, the ConfGen standard module in Glide was employed. The X-ray crystal structures of the GSK-3β complex with 2WE and CK-1δcomplexed with SR3029 (PDB ID: 4PTC and 6FAV, with X-ray resolutions of 2.71 Å and 1.40 Å, respectively) were sourced from the RCSB Protein Data Bank. The Protein Preparation Wizard in Maestro 14.0.134 was utilized to optimize the protein structure. The generated ligand conformers were subsequently docked using extra precision (XP) docking in Glide (version 6.6), within Maestro 14.0.134, and the XP Glide Score was calculated. Docking validation was carried out by docking the native ligand using the same procedure. The crystal coordinates were successfully reproduced, with an RMSD value of less than 1 Å^22^.

### Molecular Dynamics Simulation (MDS)

MDS of 500 ns each were escorted using Desmond Molecular Dynamics System version 11.8.

#### Model system generation and MD

The simulation spanned duration of 500 ns (ns) and was carried out in three main phases. Initially, the system was constructed using the System Builder module, followed by an energy minimization step to optimize the molecular arrangement and remove any unfavorable contacts. The MD simulation itself involved a three-step process: First, the XP-docked complexes were used as the starting point, and the simulation environment was set up using a predefined SPC solvent model under orthorhombic boundary conditions. Next, an energy minimization procedure was performed to reduce potential energy conflicts and provide a stable starting configuration. Finally, the MD simulation was carried out using the NPT ensemble, maintaining a temperature of 310.15 K and a pressure of 1 bar, closely mimicking real-world conditions and allowing dynamic molecular interactions with the environment^23^.

### PCA analysis

Principal Component Analysis (PCA) is a multivariate analytical method that reduces the dimensionality of complex datasets while retaining most of the original information. By converting interrelated variables into a smaller set of independent principal components, PCA facilitates the recognition of key trends and clustering patterns among compounds, protein–ligand interactions, and molecular descriptors. In the context of computational drug discovery, PCA is especially useful for evaluating molecular dynamics simulations, as it captures the dominant collective motions of protein–ligand systems and differentiates meaningful conformational states from stochastic noise^24^.

### ADMET property prediction

The designed molecules underwent in silico ADMET (Absorption, Distribution, Metabolism, Excretion, and Toxicity) property screening using the QikProp module of the Schrödinger software suite. The three-dimensional structures of sixteen ligands, previously utilized in docking studies, were analyzed to predict various physicochemical and pharmacokinetic parameters. Key features evaluated included Lipinski’s Rule of Five, polar surface area, rotatable bond count, central nervous system permeability, brain-to-blood partition coefficient, aqueous solubility, MDCK cell apparent permeability, human serum albumin binding, Caco-2 cell permeability, and the percentage of human oral absorption^25^. These parameters are essential for assessing the drug-likeness and pharmacokinetic profile of potential therapeutic candidates.

## Results and discussion

### Molecular docking scrutiny

#### Crystal structure description of GSK-3β and Tau

**Fig. 3:**
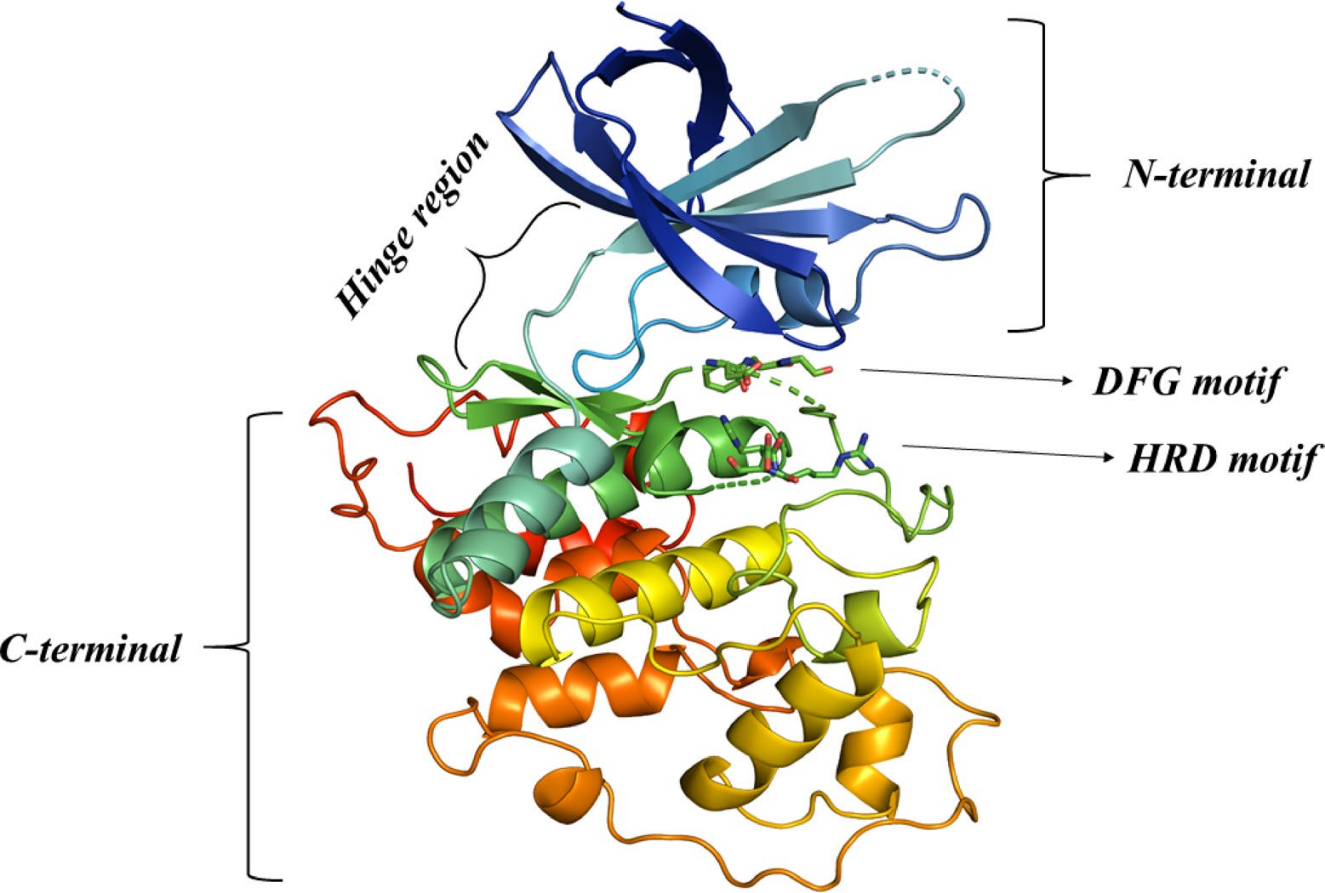
X-Ray crystal structure of GSK-3β.

Macromolecule GSK-3β is a serine/threonine kinase whose crystal structure has been determined using X-ray crystallography (PDB ID: 4PTC)^26^. This structure is divided into four primary regions: the N-terminal, C-terminal, hinge region, and activation loop. The N-terminal region, which extends from Lys36 to Leu132, features antiparallel β-strands that are stabilized by intermolecular hydrogen bonds. In contrast, the C-terminal region (from Val139-His831) is composed of a significant α-helical structure. The hinge segment (Asp133-Thr138) serves as a connector between these two regions and transitions into the flexible activation loop. An activation loop contains two critical motifs: the DFG motif, which includes Asp200, Phe201, and Gly202, and the HRD motif, consisting of His179, Arg180, and Asp181. These motifs play a crucial role in determining inhibitor selectivity. The active site for ATP binding is located between the N-terminal and C-terminal regions and incorporates the hinge region along with the DFG motif, creating an essential binding pocket for inhibitors (Fig. 3).

**Fig. 4:**
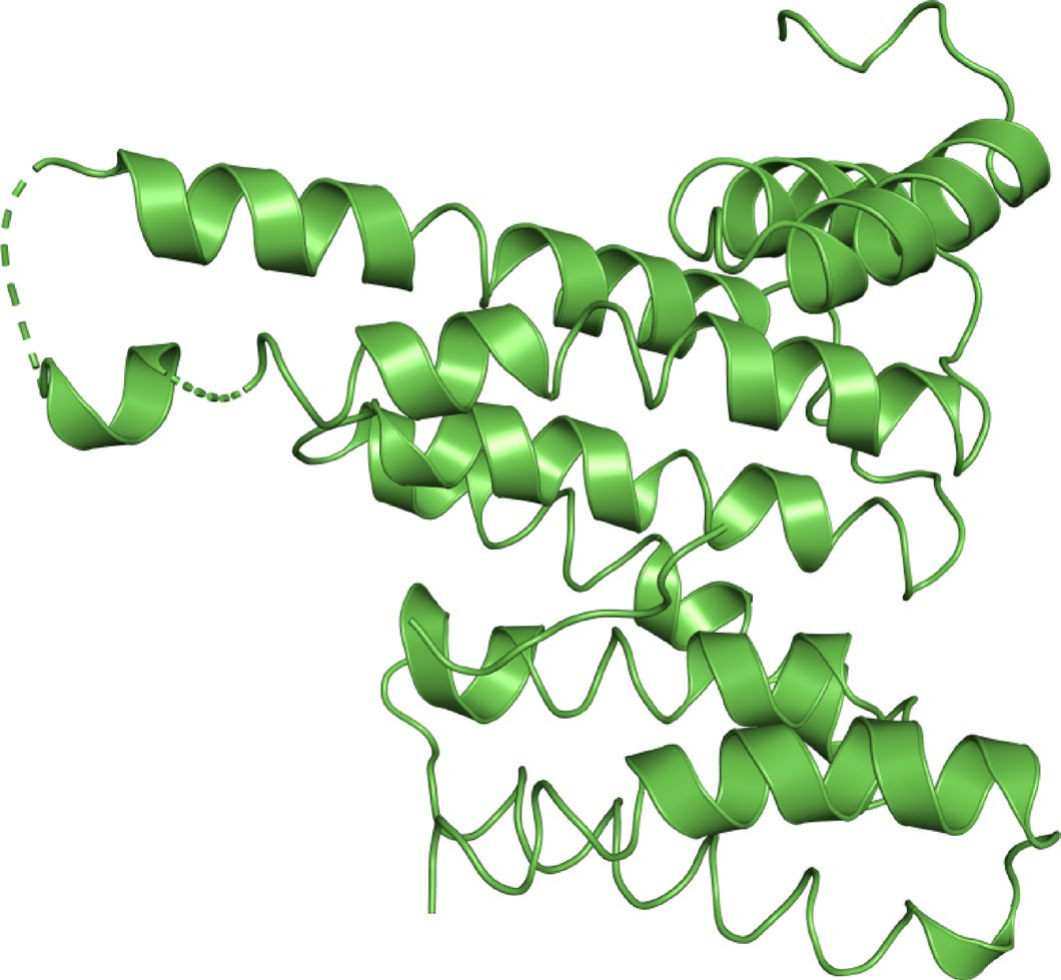
Crystal structure of Tau protein.

Tau protein is a crucial microtubule-associated protein that stabilizes microtubules in neuronal cells and exists in six major isoforms due to alternative splicing of the MAPT gene. These isoforms are classified as intrinsically disordered proteins (IDPs) and vary by the number of microtubule-binding repeats (3R or 4R) and N-terminal inserts (0N, 1N or 2N), which affects their ability to stabilize microtubules and their tendency to aggregate. The Tau structure comprises several important regions: an N-terminal region with acidic sequences that contribute to the protein’s charge, a proline-rich region that enhances flexibility, and a C-terminal region containing the microtubule-binding repeats essential for its function (PDB ID: 6FAV). Although full-length Tau has not been crystallized due to its disordered nature, researchers have successfully crystallized specific fragments, such as the core domain associated with paired helical filaments (PHFs), which show structured conformations upon aggregation and indicate a transition from disorder to order during fibril formation^27^. Additionally, Tau undergoes various post-translational modifications particularly phosphorylation, which significantly influence its structure and function; hyperphosphorylation is associated with Tau aggregation and neurodegenerative diseases like Alzheimer’s (Fig. 4).

**Fig. 5:**
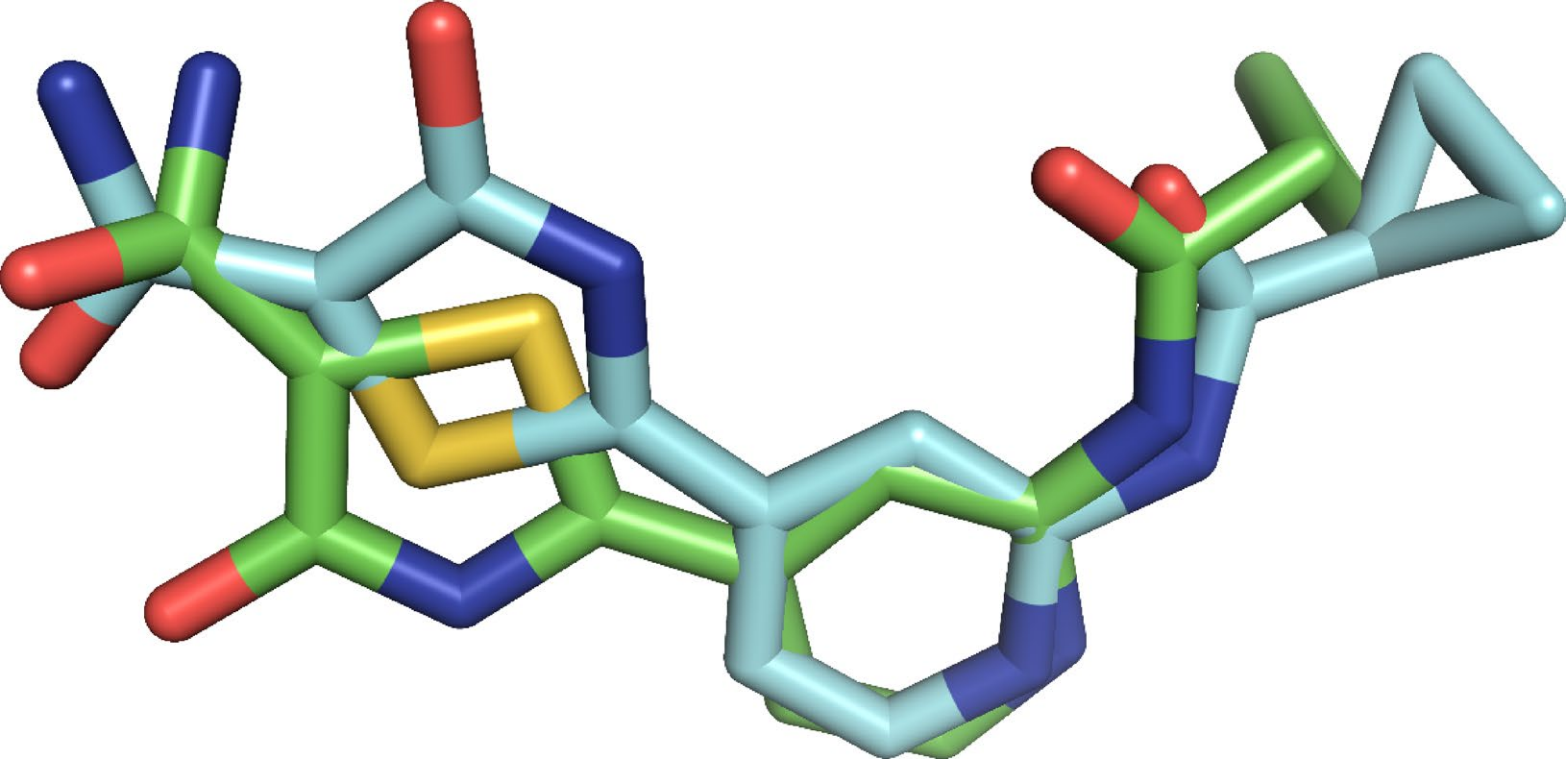
Validation of docking pose of docked conformation (green color) with co-crystal ligand (cyan color) with RMSD value of 0.32 Å.

The co-crystal ligand 2WE complexed with GSK-3β generated three polar hydrogen bond contacts with Val135, Asp200, Lys85 in the hinge region, DFG motif and catalytic arena in type one binding mode. Furthermore, crystal ligand D3Q resident in the Tau active pocket established three H bond interactions with Arg56, Arg129 and Tyr130. Validation of docking protocol was undertaken by re-docking of the aforesaid co-crystal ligand complexed with GSK-3β protein with corresponding RMSD 0.32 Å (Fig. 5). Molecular interconnections of ligands with active pocket amino acids and docking energies of the same are portrayed in Table 1.

**Table 1:** Molecular interactions and docking energies of the crafted compounds.

Sr. No	Molecule Id	Hydrogen bond interactions	Hydrophobic interactions	Docking energy (kcal.mol^-1^)
PDB ID: 4PTC (GSK-3β)				
1	Co-crystal ligand (2WE)	Lys85, Val135 and Asp200	Phe67, Val70, Ala83, Asp133, Tyr134 Leu188 and Cys199	− 6.685
2	DVK2	Lys85, Val135 and Asp200	Ile62, Phe67, Ala83, Arg141, Lys183, Leu188 and Cys199	− 7.585
3	DVK 3	Val135, Thr138 and Asp200	Ile62, Phe67, Val70, Lys85, Tyr140, Arg141 and Gln185	− 7.423
4	DVK 4	Lys85, Val135 and Asp200	Ile62, Phe67, Ala83, Arg141, Lys183, Leu188 and Cys199	− 7.896
5	DVK 5	Val135, Pro136, Thr138 and Asp200	Ile62, Phe67, Val70, Ala83 and Arg141	− 9.863
6	DVK 6	Val135, Pro136, Thr138 and Asp200	Ile62, Phe67, Ala83,Arg141and Lys182 and Cys199	− 7.333
7	DVK 7	Val135, Asn186 and Asp200	Ile62, Val70, Arg141, Lys183 and Gln185	− 8.792
8	DVK 9	Val135, Pro136, Thr138 and Asp200	Phe67, Ile62, Ala83, Arg141, Leu188, Lys183 and Cys199	− 8.824
9	DVK 11	Val135, Pro136, Glu137 and Asp200	Phe67, Val70, Ala83, Asp133, Tyr134 Lys188 and Cys199	− 6.994
10	DVK 13	Ser66, Val135, Tyr134, Gln185, Asn186 and Asp200	Ile62, Phe67, Ala83,Arg141, Lys183 and Cys199	–7.999
11	DVK 15	Val135, Asn186 and Asp200	Ile62, Phe67, Val70, Lys85, Tyr140, Arg141 and Gln185	− 6.123
12	DVK 16	Val135, Pro136, Glu137 and Asp200	Ile62, Phe67, Ala83, Arg141, Lys183 and Cys199	− 6.255
PDB ID: 6FAV (Tau protein)				
17	Co-crystal ligand (D3Q)	Arg56, Arg129 and Tyr130	Cys38, Asn42, Ser45, Lys49, Arg60, Asp126, Tyr151, Leu174, Asn175 and Asn226	− 6.789
18	DVK2	Lys49, Arg56, Arg129, Tyr130, Glu133 and Glu182	Cys38, Asn42, Ser45, Lys49, Asp126, Tyr151, Leu174, Asn175 and Asn226	− 6.812
19	DVK3	Arg129, Tyr130, Asn175 and Asn226	Cys38, Asn42, Ser45, Lys49, Arg56, Asp126, Tyr151, Leu174, Asn175 and Asn226	− 6.989
20	DVK4	Arg129, Tyr130, Asn175 and Asn226	Cys38, Asn42, Ser45, Lys49, Arg56, Asp126, Tyr151, Leu174, Asn175 and Asn226	− 7.896
21	DVK5	Lys49, Arg56, Arg60, Arg129, Tyr130, and Glu182	Cys38, Asn42, Ser45, Lys49, Asp126, Tyr151, Leu174, Asn175 and Asn226	− 7.156
22	DVK6	Arg56, Arg60, Arg129 and Glu182	Cys38, Asn42, Ser45, Lys49, Asp126, Tyr151, Leu174, Asn175 and Asn226	− 7.336
23	DVK7	Arg56, Arg60, Arg129, Tyr130 Glu182 and Asn226	Cys38, Asn42, Ser45, Lys49, Arg56, Asp126, Tyr151, Leu174, Asn175 and Asn226	− 6.766
24	DVK8	Lys49, Arg56, Arg129, Tyr130, Glu133 and Glu182	Cys38, Asn42, Ser45, Lys49, Arg56, Asp126, Tyr151, Leu174, Asn175 and Asn226	− 7.429
25	DVK9	Arg56, Lys122, Arg129 and Glu182	Cys38, Asn42, Ser45, Lys49, Arg56, Asp126, Tyr151, Leu174, Asn175 and Asn226	− 6.666
26	DVK11	Arg56, Lys122, Arg129 and Glu182	Cys38, Asn42, Ser45, Lys49, Arg56, Asp126, Tyr151, Leu174, Asn175 and Asn226	–8.994
27	DVK12	Lys49, Arg56, Arg60, Glu122, Arg129, Tyr130, Glu133, Asn175 and Asn226	Cys38, Asn42, Ser45, Lys49, Arg56, Lus122, Asp126, Tyr151, Leu174, Asn175 and Asn226	− 8.056
28	DVK14	Arg56, Lys122, Arg129 and Glu182	Cys38, Asn42, Ser45, Lys49, Arg56, Asp126, Tyr151, Leu174, Asn175 and Asn226	− 7.267
29	DVK16	Arg56, Glu133, Lys140 and Asn175	Cys38, Asn42, Ser45, Lys49, Arg56, Asp126, Tyr151, Leu174, Asn175 and Asn226	− 7.156

#### Molecular contacts of crafted molecules with enzyme GSK-3β

**Fig. 6:**
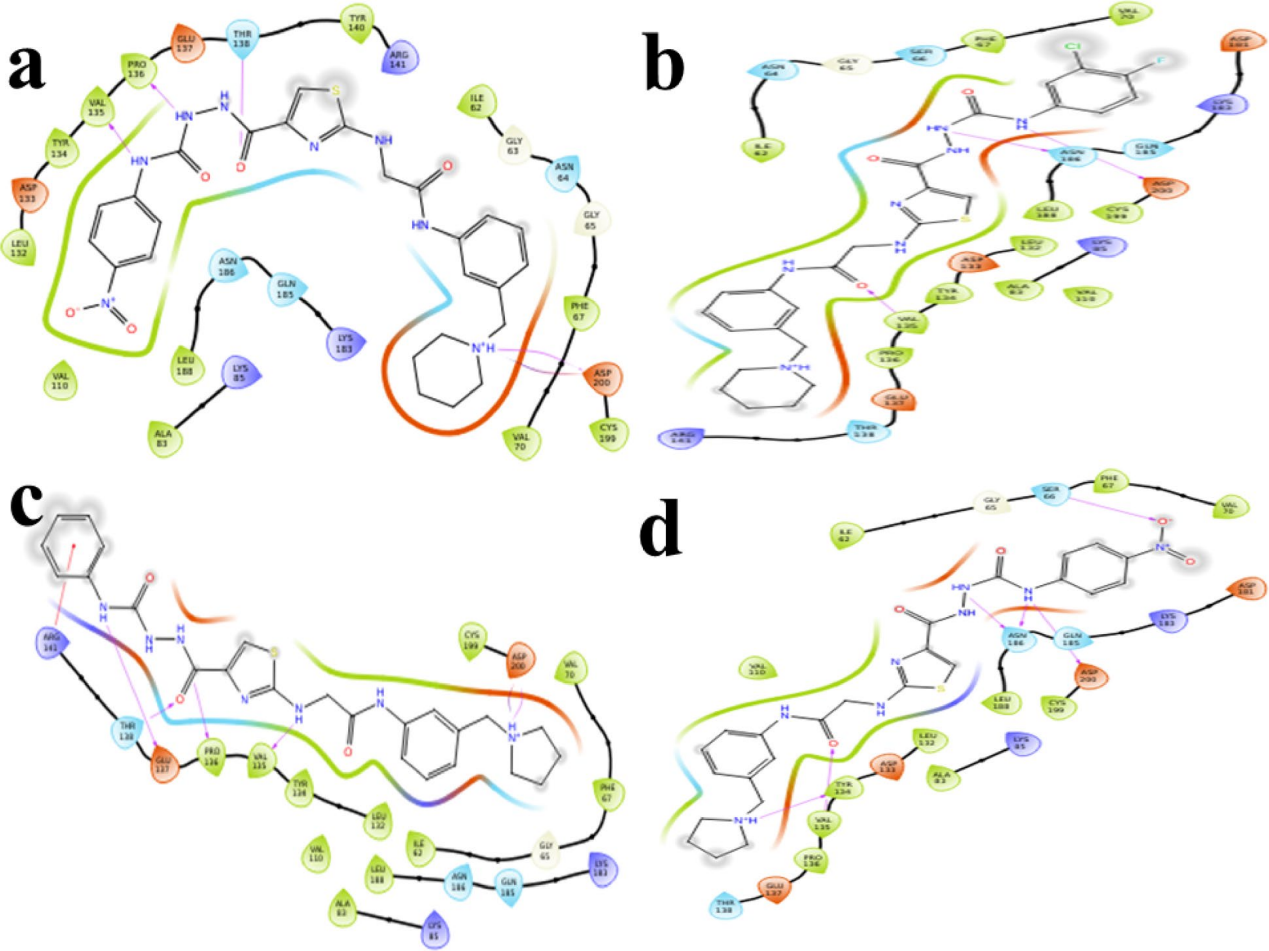
Docked complex of (**a**) DVK5, (**b**) DVK7, (**c**) DVK9, (**d**) DVK13 withGSK-3β.

Compound **DKV5** with excellent docking energy **– 9.863 kcal/mol** inhabited the active pocket of an enzyme. The NH and carbonyl oxygen of hydrazide carboxyl functional group established three polar hydrogen bond interactions with Val135, Pro136 and Thr138 in the hinge region. Similarly, protonated NH of piperidine ring developed two H bond interconnections with Asp200 in the allosteric DFG motif of the kinase (Fig. 6a).

Molecule entitled **DKV7** was appropriately anchored in the binding groove of macromolecule where the carbonyl oxygen of the acetamide linker generated a polar hydrogen bond interconnection with Val135 in the hinge region. NH of hydrazide carbonyl group established two H bond interactions with Asp200 and Asn186 in DFG and side chain motifs. Furthermore, heterocyclic ring thiazole was engrossed in the hydrophobic cavity and formed π-alkyl contacts with Val70, Ala83, Lys85, Leu132 and Cys199. Similarly, the aromatic ring attached to an acetamide chain generated hydrophobic π-π stack interlinkage with Tyr134 in the lipophilic cavity of hinge arena with corresponding docking energy **-8.792 kcal.mol**^**-1**^ as displayed in Fig. 6b.

Compound titled **DKV9** with docking energy of **-8.824 kcal/mol** was affixed in an active cavity of the protein. The NH of acetamide, carbonyl oxygen and NH of hydrazide urea functional group established four polar hydrogen bond contacts in the hinge area. The protonated nitrogen of pyrrolidine fragment formed two H bond interactions with Asp200 in DFG motif of kinase. Moreover, phenyl ring flanked to hydrazide carboxyl group generated electrostatic π-cation interconnection with Arg143 as presented in Fig. 6c.

Molecule **DKV13** was well fixed in an active groove of the GSK-3β where oxygen of acetamide, NH of the pyrrolidine ring engendered two hydrogen bond linkages with Val135 and Thr134 in the hinge demesne. Nitrogen of hydrazide carboxyamide functional group established three H bond interactions with Asp200, Asn186 in the DFG and side chain arenas. A nitro group attached on the *para* position of the phenyl ring generated hydrogen bond interconnection with Ser66 in the N-terminal region with corresponding docking energy **–7.999 kcal/mol** (Fig. 6d).

#### Molecular interactions of designed compounds with Tau protein

**Fig. 7:**
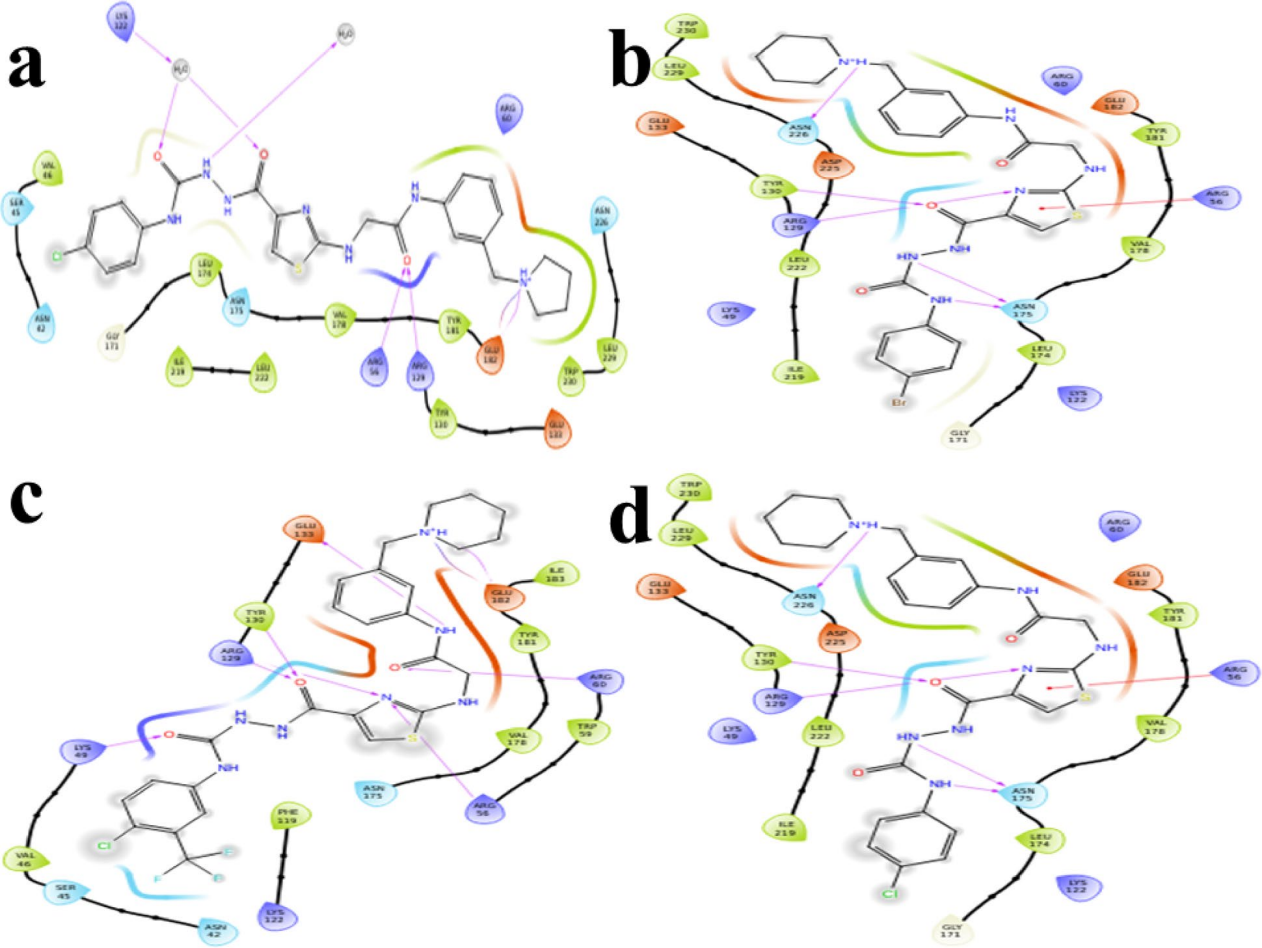
Docked complex of (**a**) DVK11, (**b**) DVK4, (**c**) DVK8, (**d**) DVK3 with Tau protein.

Compound titled **DKV11** occupied the binding sector of protein with corresponding docking energy **–8.994 kcal/mol**. Carbonyl oxygen of an acetamide fraction (linker 1) generated two polar hydrogen bond interactions with Arg56 and Arg129 similarly, two carbonyls of hydrazide carboxymide (linker 2) established three water mediate H bond interconnections with Lys322. The protonated nitrogen of the pyrrolidine (Tail part) formed two H bond interlinkages with Glu182 as portrayed in Fig. 7a.

Molecule **DKV4** resided appropriately in an active groove of the enzyme Tau where, carbonyl oxygen and NH of the semi carbazide generated three polar hydrogen bond interlinkages with Tyr130 and Asn175. Moreover, 3^rd^ nitrogen of thiazole ring and protonated piperidine entrenched H bond contacts with Arg129 and Asn226. A central heterocyclic thiazole ring formed electrostatic π-cation stack linkage with Arg56 with corresponding docking energy of **-7.896 kcal.mol**^**-1**^as exhibited in Fig. 7b.

Compound designated **DVK8** abided in the binding area of a macromolecule with associated docking energy of **-7.429 kcal/mol**. The oxygen and nitrogen of an acetamide part (linker 1) of the compound formed two H bond interconnections with Arg60 and Glu133, NH of piperidine established hydrogen bond interactions Glu182. Similarly, first carbonyl oxygen of the semi carbazide (linker 2) and 3^rd^ N of thiazole generated four hydrogen bond interactions with Arg129, Thr130 and Arg56. Second carbonyl functional group generated hydrogen bond linkage with Lys49; the head part of the molecule i.e. 3-trifluoromethyl-4-chloro phenyl ring occupied hydrophobic pocket which was formed by the acids namely Asn42, Ser45, Val46, Phe119 and Lys122 as displayed in Fig. 7c.

Molecule entitled **DVK3** anchored in the active arena of protein where the carbonyl oxygen, NH of semi carbazide group and third nitrogen of thiazole moiety established five polar hydrogen bond interactions with Arg129, Tyr130 and Asn175. Heterocyclic piperidine with protonated nitrogen developed unit H bond contact with Asn226. Furthermore, the -4Cl phenyl ring, central thiazole and phenyl ring attached to acetamide linker generated electrostatic π-cation and π-anion interlinkages with Glu35, Cys38 with corresponding docking energy of **-6.989 kcal/mol** as picturized in Fig. 7d. All the crafted compounds depicted akin, molecular interlinkages as that of the co-crystal ligands adhering to both the enzymes discretely.

### Molecular Dynamics Simulation (MDS) analysis

MDS is a computational technique that is employed to validate the docking outcomes where the solidity of protein and its corresponding docked complex is assessed in each time frame. Quality of the simulation was examined using factors namely RMSD, RMSF and protein–ligand interlinkage analyses. Based on the molecular contacts and docking energy profiles, compounds **DKV5** and **DKV11** were nominated for detailed evaluation on GSK-3β and Tau proteins over a 500 ns timescale.

#### RMSD plots

##### Protein

**Fig. 8:**
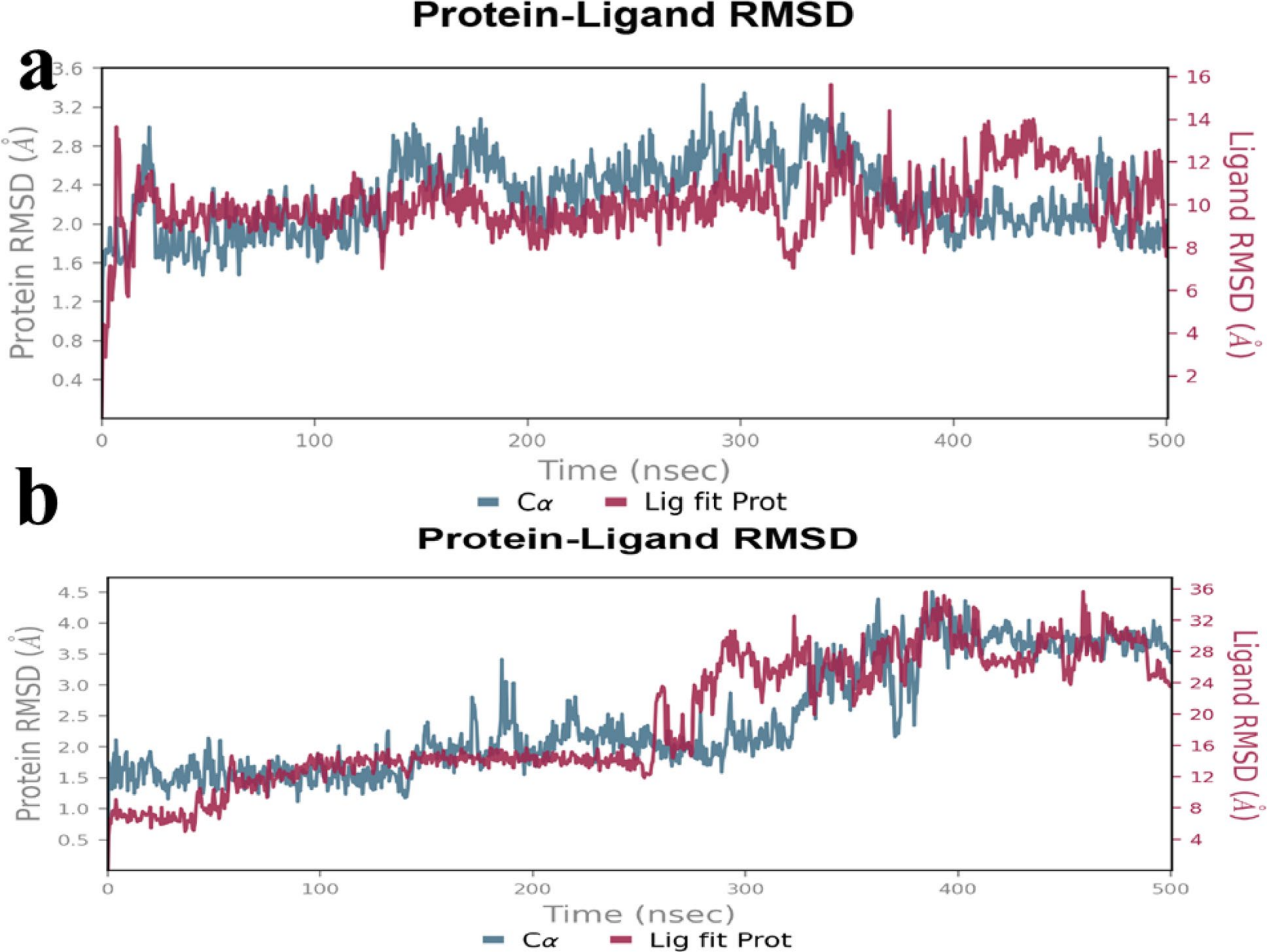
RMSD plots of (**a**) GSK-3β DVK5, (**b**) Tau DVK11.

The RMSD graph stated an average alteration in the expulsion of an election of atoms for a target concerning the reference framework. It delivers a visual portrayal of alterations in the protein structure over designated time duration during the simulation. At an initial time, frame of MD RMSD of protein GSK-3β was observed at 1.6 Å with sudden rise till 3 Å from 1 to 25 ns. A fall in the spikes were noticed till 1.5 Å which was maintained constant till 140 ns, further a sudden rise till 2.9 Å was spotted in the array of 150 – 200 ns and similar sudden spikes were recognized from 300 to 400 ns after which with small episodes of rise and fall in projection it equilibrated to 1.8 Å till end of the dynamic trajectory (Fig. 8a).

Akin, to GSK-3β the RMSD of Tau was initiated at 1.5 Å which further rose to 2.2 Å with a downfall of the hill to 1 Å till 50 ns. A sudden equilibrium in the structure of the enzyme was spotted from 51 – 200 ns with small episodes of rise and fall in RMSD projections between 3 – 2 Å. Moreover, in the scrutiny spikes a sudden upliftment in RMSD spikes were noticed till 3.5 Å from 310 to 450 ns. Protein gained conformational solidity till end of dynamic ambit as picturized in Fig. 8b. The protein–ligand complexes (GSK-3β DVK12 and CK-1δ DVK14) demonstrated a concrete conformational solidity of the molecules within the enzymes’ binding pocket, as projected in the graph analysis (Fig. 8a, b).

### RMSF graph evaluation

#### Macromolecule

**Fig. 9:**
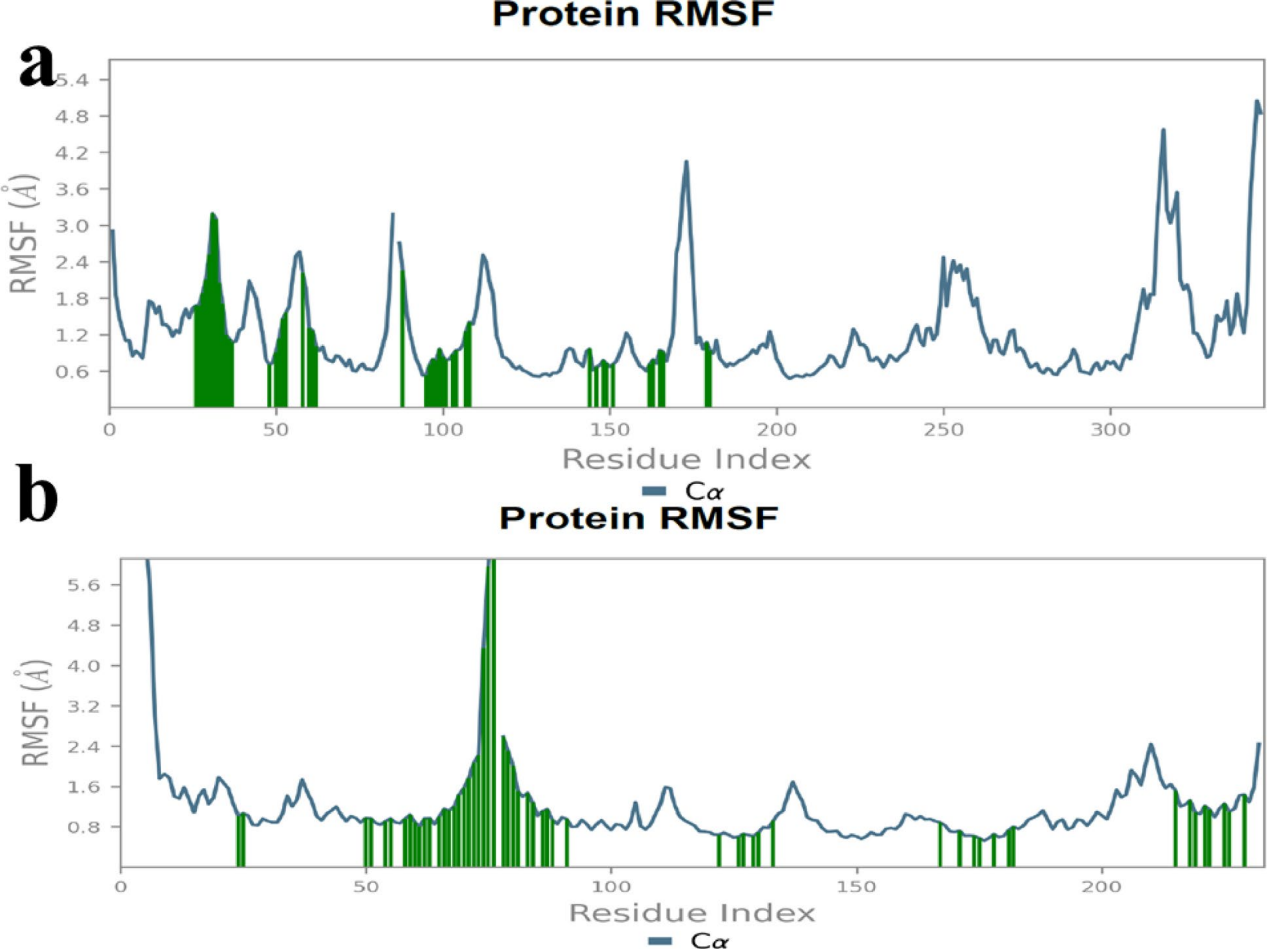
RMSF plots of (**a**) GSK-3 DVK5, (**b**) Tau protein DVK11, the green colored lines represent the favorable contacts.

RMSF is an essential metric for assessing the quality of molecular dynamics simulations, as it quantifies the average fluctuation of amino acid residues over time relative to their initial positions. RMSF provides insight into the degree of deviation of amino acid positions throughout the simulation. Figures 9a, b demonstrated the RMSF values for the amino acids in GSK-3β and CK-1δ. The α-helical regions underlined in magenta, β-sheets in green and the active site residues, which interact with inhibitors, are marked in red color. Notably, greater fluctuations were observed in the terminal amino acids, which are located farther from the binding site. Minimal and inconsequential conformational changes were detected in both the active site and the protein chain. In general, the fluctuations of residues near the inhibitors remained stable, staying below 2.0 Å, suggesting that the interactions were stable throughout the simulation.

##### Ligands

**Fig. 10:**
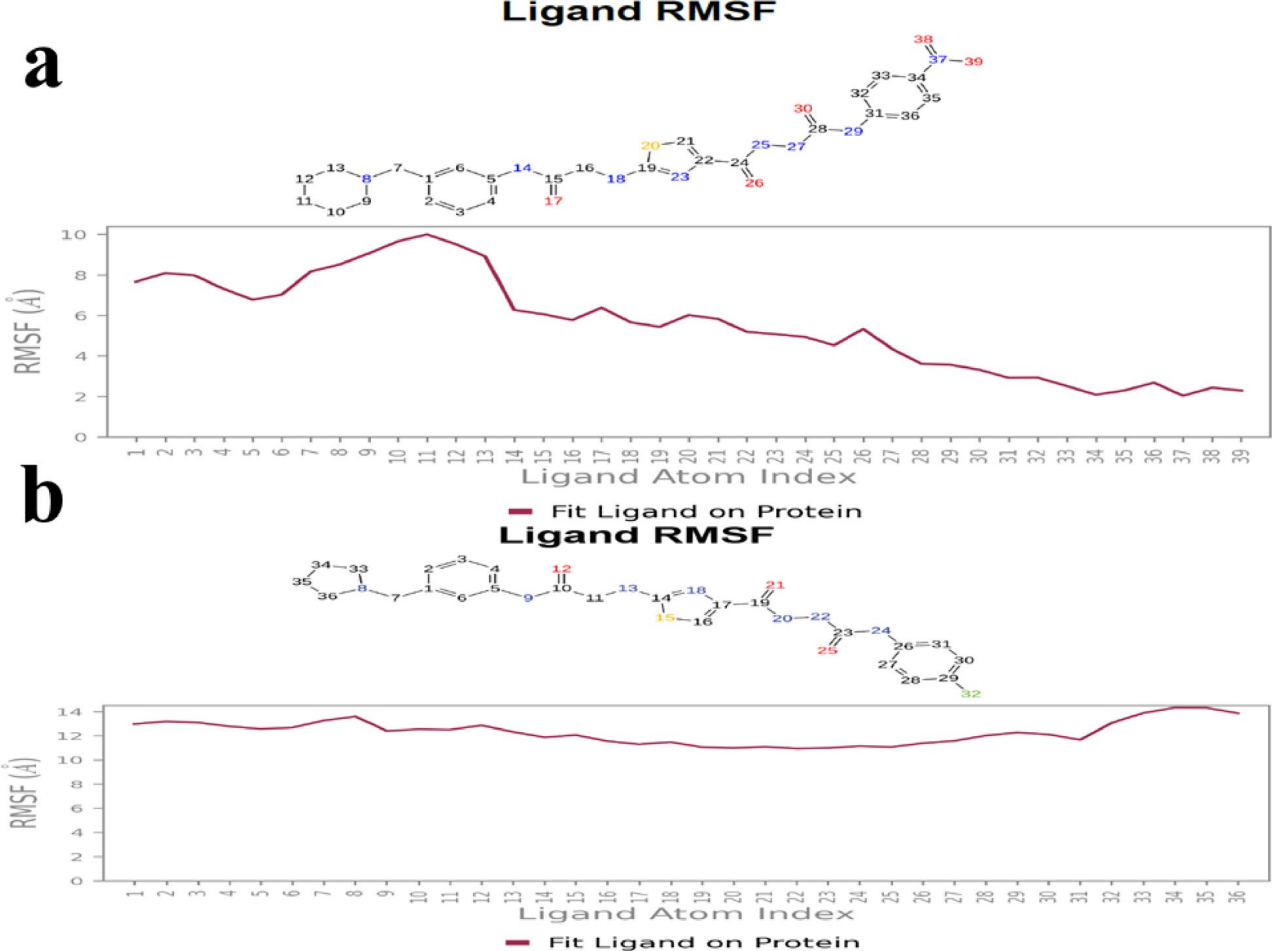
RMSF plots of (**a**) DVK5, (**b**) DVK11.

Molecules **DKV5** and **DKV11 **demonstrated minimal fluctuation was observed over the 500 ns timescale, with functional group such as methyl exhibiting higher fluctuations compared to other structural features of the molecules under analysis, as shown in Figs. 10a, b.

#### Protein–ligand / ligand protein interaction plots

**Fig. 11:**
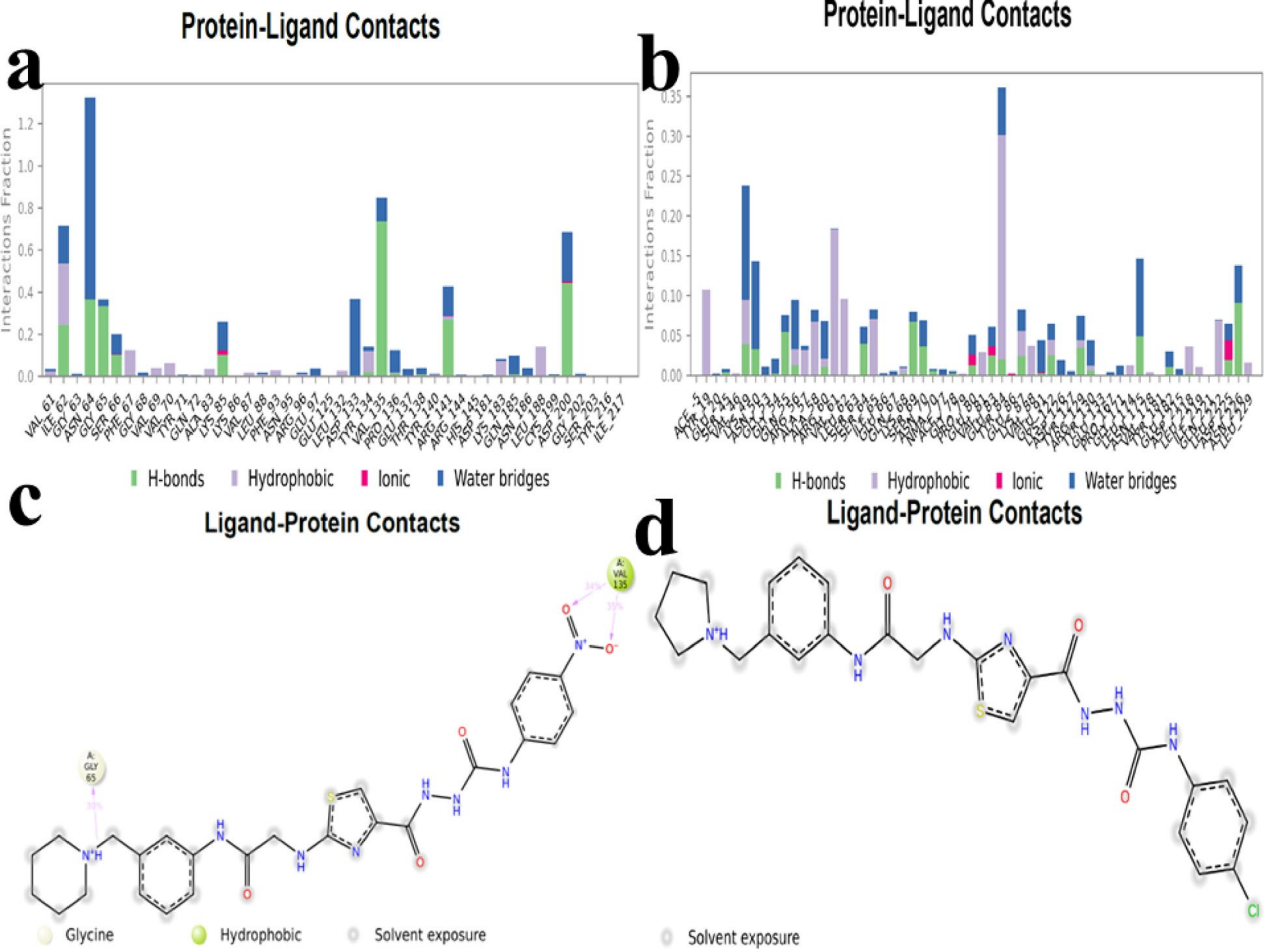
Molecular contacts of (**a**, **c**) GSK-3β DVK5; (**b**, **d**) Tau DVK11 complexes in MD ambit.

A key aspect of evaluating the stability of a protein–ligand complex is examining the molecular interactions between the ligand and the active site amino acids of the enzyme. Several types of interactions, including electrostatic, hydrogen bonding, hydrophobic, water bridges, and polar ionic interactions, are considered. The protein–ligand interaction histogram revealed that hydrogen bonds and water bridge interactions accounted for a higher percentage compared to hydrophobic and electrostatic interactions. In the time amid of MD scrutiny **DVK5** with **GSK-3β** divulged polar hydrogen bond interactions with Val135 (34 and 35%). Kindred, **DVK11** with **Tau** portrayed hydrophobic van der Walls interactions with Ala36 as picturized in Fig. 11a-d.

### Molecular dynamics scrutiny of co-crystal ligands (reference standard)

**Fig. 12:**
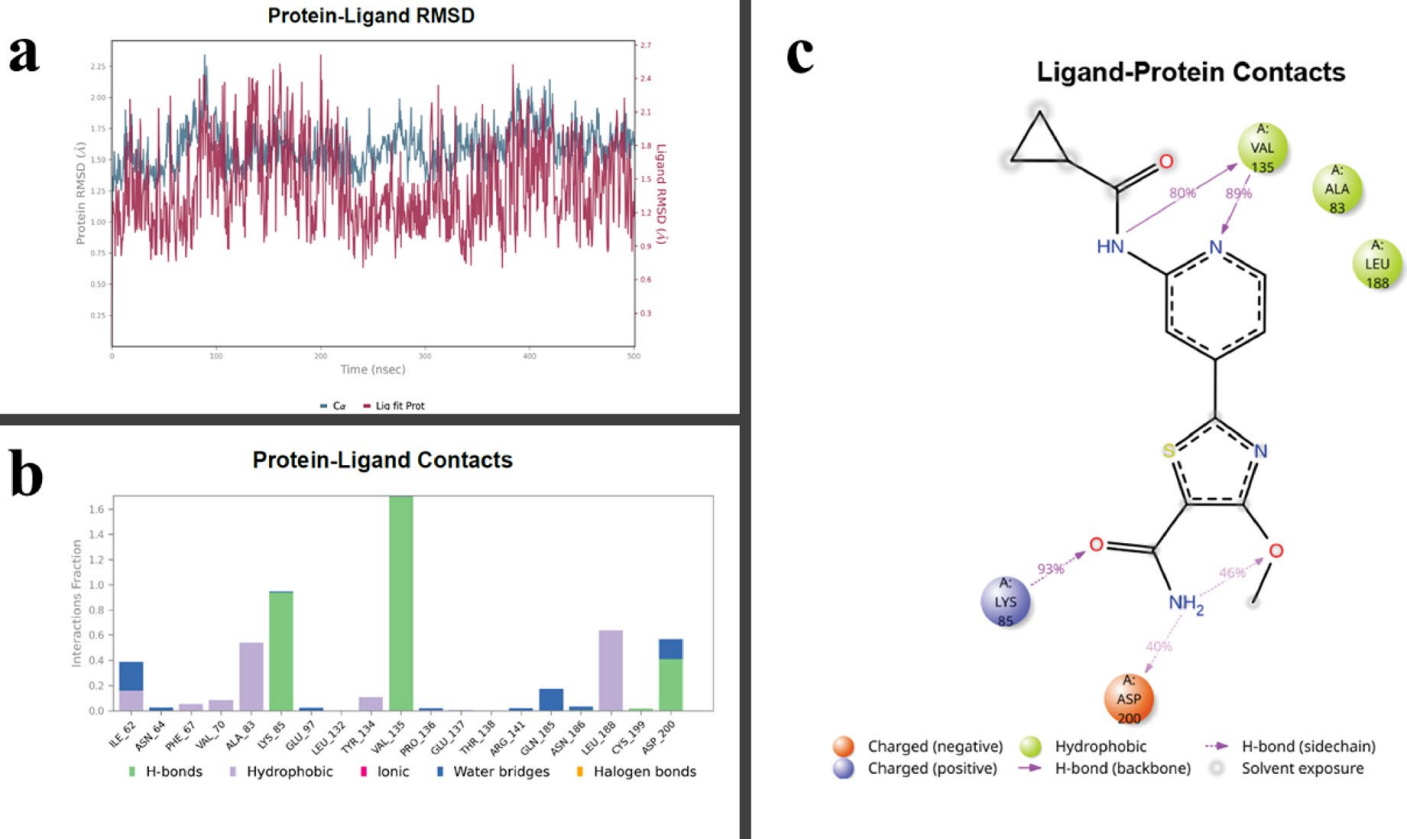
MD outcomes of co-crystal ligand 2WE of the PDB 4PTC (**a**) RMSD plots; (**b**, **c**) Protein ligand interaction graphs.

MD results of the in-housed ligand 2WE with PDB 4PTC are displayed in Fig. 12a. RMSD graph of protein ligand complex portrayed to be stable throughout the 500 ns time frame. There was no drastic elevation in the spikes noticed which stated the solidity of inhibitor in the binding pocket of GSK-3β. Molecular interconnections of 2WE exhibited that N of amide group and pyridine ring established hydrogen bond interaction with Val135 in the hinge area and carbonyl oxygen and nitrogen of amide functionality of primary amide flanked on 2^nd^ position developed interaction with Lys86 and Asp200 in the catalytic and DFG motif of GSK-3β.

**Fig. 13:**
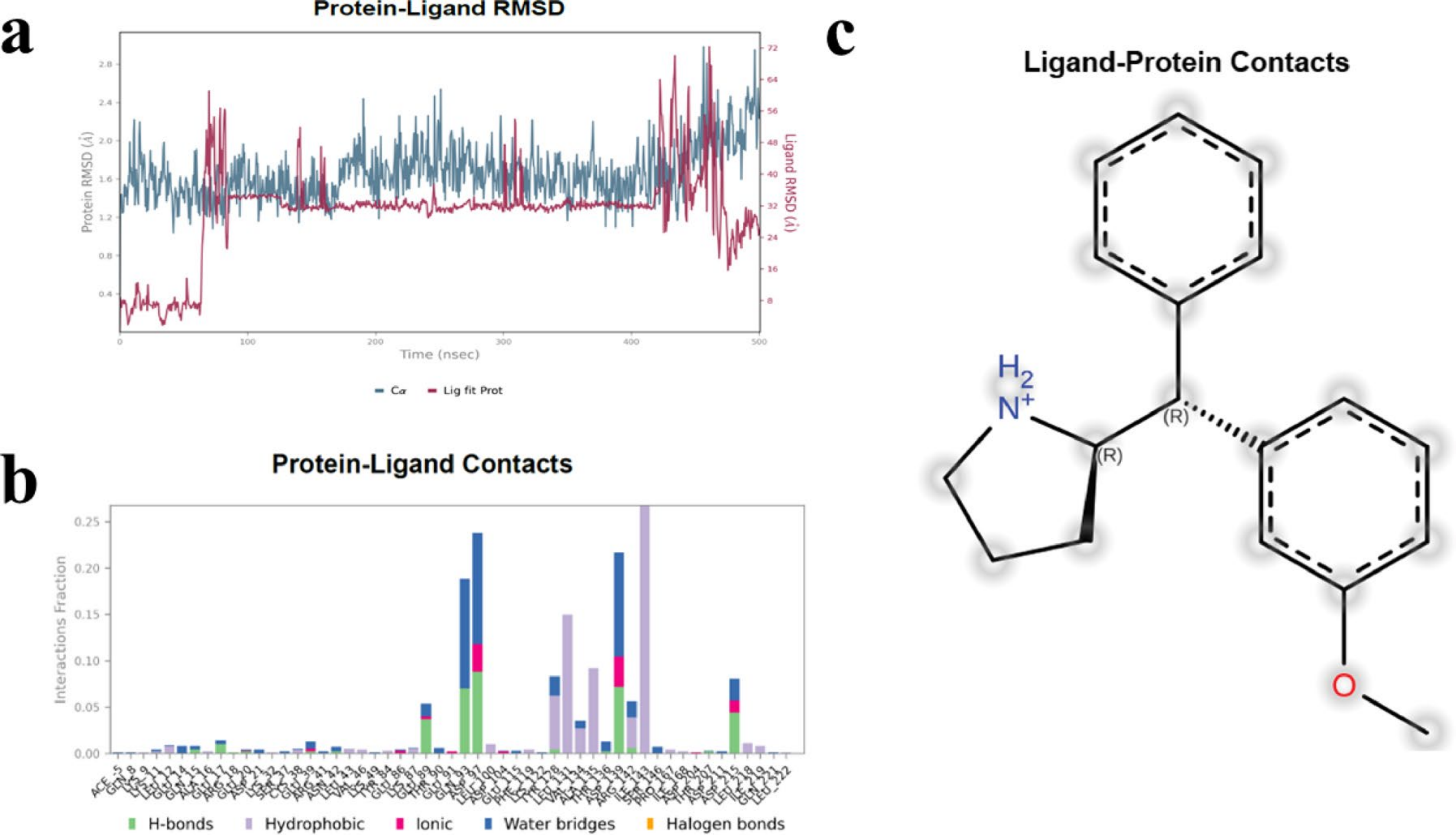
MD results of co-crystal ligand D3Q of the PDB 6FAV (**a**) RMSD plots; (**b**, **c**) Protein ligand interaction graphs.

MD outputs of the co-crystal ligand D3Q with PDB 6FAV are portrayed in Fig. 13a. RMSD plot of protein ligand complex showed to a mediocre stability throughout the 500 ns time frame. There were some escalations in the RMSD spikes noticed which stated the moderate solidity of inhibitor in the binding cavity of Tau. Molecular interactions of D3Q stated hydrophobic and ionic contacts with the active site of 6FAV.

### PCA scrutiny

**Fig. 14:**
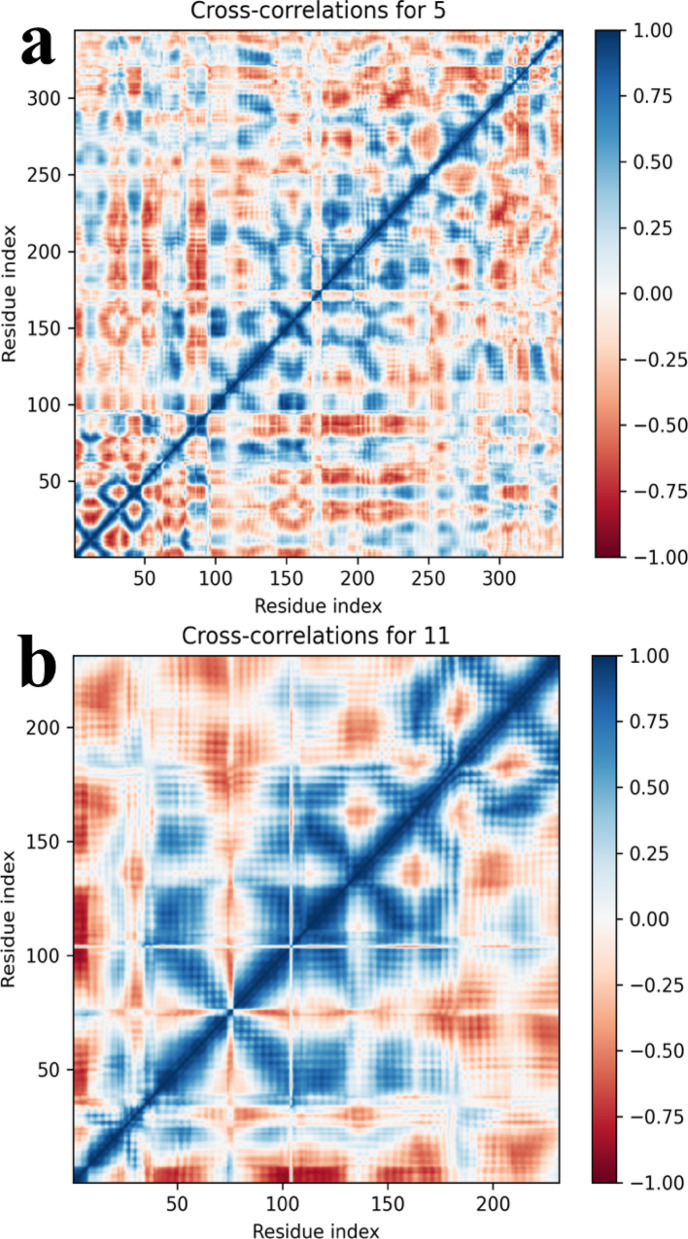
PCA plots of (**a**) GSK-3β DVK5 (**b**) Tau DVK11.

The cross-correlation assessment of the GSK-3β and Tau systems demonstrates that ligand association predominantly promotes positive residue correlations, indicative of synchronized motions and increased structural stability within the complexes. Concurrently, the presence of anti-correlated movements suggests that essential conformational flexibility is preserved, enabling induced-fit interactions and facilitating allosteric communication. Enhanced positive correlations in the vicinity of the active site further indicate stabilization of crucial binding residues, which is likely to contribute to stronger binding interactions and extended ligand residence. From a translational standpoint, these findings highlight **DVK5 and DVK11** as viable lead scaffolds, as they maintain an effective balance between conformational stability and dynamic adaptability. This dual characteristic is particularly relevant in structure-based drug design, where achieving optimal kinase inhibition and regulating Tau-related pathological mechanisms require both rigidity for stable binding and flexibility for functional responsiveness (Fig. 14).

### In silico ADMET forecast

Schrödinger’s QikProp module has proven to be an essential tool for reliably predicting the ADMET properties of designed molecules. Table 2 provides a summary of the evaluated molecules along with their respective properties. For this analysis, Lipinski’s Rule of Five was applied, considering factors such as molecular weight, polar surface area, the number of hydrogen bond donors and acceptors, as well as the calculated octanol–water partition coefficients. Series of molecules analyzed adhered to the criteria specified by Lipinski’s Rule of Five. Specifically, they each had fewer than 5 hydrogen bond donors, fewer than 10 hydrogen bond acceptors, and a QPlogP value under 5. The molecular weights of the molecules ranged from 493.582 to 610.053 daltons, while the Toplogical Polar Surface Area (TPSA) values spanned from 147.49 to 196.593 Å^2^, all of which were within the acceptable limits of LR5. The oral absorption percentages of the designed molecules ranged from 26.24 to 75.175%, with compound **DVK10** exhibiting the highest oral absorption at 75.175%. The toxicity predictions revealed that all the designed molecules did not forecast any toxicity. The reference standard 2WE an GSK-3β inhibitor displayed oral absorption of 80.596% and no AEMS toxicity as compared to designed molecules. Overall, all the designed molecules met the ADMET criteria with satisfactory safety and efficacy profiles.

**Table 2:** In silico ADMET properties forecast of designed molecules.

Lipinski Rule of 5							Jorgensen’s Rule of Three				*In Silico* Predicted ADMET					
Compd ID	Mol. Wt	Donor HB	Accpt. HB	QPlogP o/w	PSA	Violation	QP logS	QPP Caco	#metab	Violation	QP logBB	QPP MDCK	QP logKhsa	SASA	% Human Oral Absorption	Toxicity
DVK1	507.609	2.75	8.75	3.432	151.454	1	− 6.001	20.679	6	2	− 1.978	22.248	0.402	909.76	57.63	Nontoxic
DVK2	525.6	2.75	8.75	3.667	151.416	1	− 6.353	20.885	5	2	− 1.87	40.709	0.441	918.302	59.079	Nontoxic
DVK3	542.054	2.75	8.75	3.958	151.458	1	− 6.502	25.339	5	1	− 1.665	68.627	0.507	920.995	62.284	Nontoxic
DVK4	586.505	2.75	8.75	4.033	151.437	1	− 6.606	25.565	5	1	− 1.653	74.527	0.528	925.52	62.794	Nontoxic
DVK5	552.607	2.75	9.75	2.776	196.593	2	− 5.882	3.166	6	2	− 3.105	2.938	0.345	933.136	26.24	Nontoxic
DVK6	575.608	2.75	8.75	3.274	149.926	1	− 3.77	28.173	5	0	− 1.023	80.299	0.309	759.382	59.104	Nontoxic
DVK7	560.045	2.75	8.75	4.161	152.013	1	− 6.707	23.263	5	1	− 1.582	108.024	0.554	922.598	62.811	Nontoxic
DVK8	610.053	2.75	8.75	3.681	149.87	1	− 4.425	23.759	6	0	− 1.149	75.104	0.47	800.178	60.165	Nontoxic
DVK9	493.582	2.75	8.75	2.764	145.887	0	− 3.841	27.073	6	0	− 1.36	34.367	0.147	773.584	68.77	Nontoxic
DVK10	493.582	2.75	8.75	2.838	147.531	0	− 3.627	58.4	6	0	− 0.98	67.929	0.112	745.303	75.175	Nontoxic
DVK11	528.027	2.75	8.75	3.26	145.78	1	− 4.648	42.474	5	0	− 1.068	102.513	0.241	782.368	62.218	Nontoxic
DVK12	572.478	2.75	8.75	3.399	147.49	1	− 4.483	58.915	5	0	− 0.84	181.94	0.234	777.002	65.571	Nontoxic
DVK13	538.58	2.75	9.75	2.249	192.278	2	− 3.163	7.998	6	1	− 1.831	9.724	0.062	766.408	30.359	Nontoxic
DVK14	561.581	2.75	8.75	4.157	151.924	1	− 6.686	26.926	5	1	− 1.502	131.887	0.519	919.278	63.926	Nontoxic
DVK15	546.018	2.75	8.75	3.822	151.992	1	− 6.359	23.614	5	1	− 1.588	102.217	0.417	902.507	60.941	Nontoxic
DVK16	596.026	2.75	8.75	3.231	145.782	1	− 3.91	36.879	6	0	− 0.774	207.115	0.21	738.162	60.947	Nontoxic

## Conclusion

AD is a prevalent and challenging neurodegenerative disorder, and current treatments have shown limited effectiveness, highlighting the urgent need for novel therapeutic approaches. This study aimed to design drug-like molecules that not only offer enhanced efficacy but also minimize the risk of side effects. To achieve this, the researchers conducted detailed molecular docking studies targeting both GSK-3β and Tau enzymes. The findings revealed that compounds **DVK5** and **DVK11** exhibited favorable interactions within the active sites of these proteins, with critical amino acid interactions and promising docking energies of **– 9.863** and **– 8.994 kcal/mol**. Furthermore, molecular dynamics simulations were performed to assess the stability of the **DVK5 and DVK11** complexes within the active pockets of GSK-3β and Tau. The outcomes demonstrated that these protein–ligand complexes maintained stable interactions throughout the 500 ns simulation trajectory. In silico ADMET analysis showed that **DVK10** had an outstanding human oral absorption rate of **75.175%**, outperforming other compounds in the series. Overall, the in silico studies strongly support the potential of these molecules to effectively inhibit both Tau and GSK-3β, providing a promising foundation for the development of new lead compounds aimed at addressing the challenging effects of AD.

## Data Availability

All data generated or analyzed during this study are included in this published article.” as there are no patient trials in this manuscript.
